# Correlation between the severity of COVID-19 infection and the presence of oropharyngeal candidiasis: a cross-sectional study in Al-Diwaniyah Hospital, Iraq

**DOI:** 10.11604/pamj.2025.50.25.43995

**Published:** 2025-01-13

**Authors:** Laila Jasim Shaebth, Hassan Hachim Naser, Louhichi Nacim

**Affiliations:** 1 Department of Biology, Faculty of Science, University of Sfax, Sfax, Tunisia,; 2 Technical Institute of Al-Diwaniyah, Al-Furat Al-Awsat Technical University, Diwaniyah, Iraq,; 3 Department of Microbiology, College of Veterinary Medicine, University of Al-Qadisiyah, Al-Qadisiyah, Iraq,; 4 Laboratory of Molecular and Cellular Screening Processes, Center of Biotechnology of Sfax, University of Sfax, Road of Sidi Mansour Km 6, P.O. Box 1177, Sfax 3018, Tunisia

**Keywords:** COVID-19, candida albicans, C-reactive protein, interleukin-6, oropharyngeal

## Abstract

**Introduction:**

oropharyngeal candidiasis, commonly known as oral thrush, is a fungal infection caused by candida species, especially Candida albicans. With the onset of COVID-19, concerns have arisen about a possible link between the severity of COVID-19 infections and the presence of oropharyngeal candidiasis in patients. The aim to study the frequency of oropharyngeal candidiasis in COVID-19 patients and identify associated risk factors.

**Methods:**

a cross-sectional study involving 100 COVID-19 patients was conducted at Al-Diwaniyah Hospital in Iraq. Samples were collected using blood tests and oropharyngeal tampons to diagnose oropharyngeal candidiasis. The mean age of the study population was 55.3 years (SD±12.4), with 60% male and 40% female. The data was analyzed using IBM SPSS software version 22, focusing on the correlation between the severity of COVID-19 and the occurrence of candidiasis.

**Results:**

among the 100 patients, 30 (30%) were diagnosed with oropharyngeal candidiasis. In patients with severe COVID-19 (45%) symptoms, the prevalence was significantly higher than in patients with mild (15%) and moderate (25%) symptoms (p=0.002). In addition, patients with candidiasis (CRP: 12.5 mg/l) had significantly higher mean levels of C-reactive protein (CRP: 12.5 mg/l) than patients without candidiasis (CRP: 6.8 mg/l, p < 0.001). Other risk factors identified included long-term hospitalization (mean duration of patients with candidiasis 12 days vs. 7 days without patients, p=0.005) and use of corticosteroids (70% of patients with candidiasis vs. 40% without, p=0.01).

**Conclusion:**

the results show that oropharyngeal candidiasis is common among COVID-19 patients, especially those with severe infections, with a prevalence rate of 45%. The study emphasizes the importance of monitoring fungal infections in COVID-19 patients and the need for early diagnosis and treatment to improve patient outcomes. These findings are supported by the significant differences in prevalence and inflammatory markers observed in the study.

## Introduction

The coronavirus family consists of large, enclosed, single-stranded RNA viruses that range in diameter from 65 to 125 nanometers. The four subfamilies of coronaviruses are denoted by the letters α, β, γ, and δ. Some people infected with alpha lineage coronaviruses show no symptoms whatsoever. The flip side is that beta-linage is linked to an increased risk of mortality and major disease [1]. One example is the 2009 H1N1 pandemic, caused by the beta coronavirus; another is the Middle East respiratory syndrome coronavirus, which was caused by the H5N1 influenza-A virus.

A greater likelihood of ARDS and ALI has been linked to them [2]. An encapsulated single-stranded RNA coronavirus, COVID-19 (SARS-CoV-2) shares around 80% sequence similarity with SARS-CoV. Symptoms caused by COVID-19 can range from moderate to severe, depending on the person infected. A high fever, dry cough, wheezing, and severe exhaustion are typical signs of illness [3]. Reduced lung compliance and alveolar collapse are symptoms of COVID-19 ALI, marked by low levels of pulmonary surfactant protein and intra-alveolar fibrin buildup. Lung fibrosis develops when surfactant levels are low and fibrin is abundant inside the alveoli [4].

The presence and severity of ALI determine the prognosis for the patient. Patients in critical care improve outcomes and reduce mortality when given corticosteroids early during a cytokine storm [5]. It has been found that the likelihood of mortality is significantly increased when there is a co-infection [6]. In patients with COVID-19 who are receiving invasive or non-invasive ventilation in an intensive care unit, superinfection with bacteria and fungi has been documented. Some examples of clinical observations are blood counts and imaging findings, and these can be further subdivided into patient demographics.

However, the criteria used to predict the severe course of the COVID-19 disease are unclear. Individual treatment programs are needed to meet the unique needs of each patient [7]. Oropharyngeal candidiasis (OPC), caused by colonization of the oral mucosa by Candida species, may be a source of morbidity in patients with severe coronavirus disease 2019 (COVID-19), according to our center's experience in treating these patients. More than 80% of oral candidiasis cases are caused by *Candida albicans*. However, evidence suggests that candida species other than *C. albicans* play a role in this disease Finding etiological agents and early detection of oropharyngeal candidiasis (OPC) are crucial in optimizing the treatment and prognosis of COVID-19 patients. Despite being the most common mucocutaneous mycosis of the mouth, it is unclear with what frequency severe COVID-19 patients may develop oropharyngeal candidiasis (OPC). The COVID-19 pandemic has had a significant impact on global health, causing an increase in various opportunistic infections among those affected. One such infection is oropharyngeal candidiasis, commonly known as oral thrush, caused by candida species especially *Candida albicans*. Patients with COVID-19, especially those with severe symptoms, have an increased risk of developing fungal infections due to factors such as immunosuppression, prolonged hospitalization and the use of corticosteroids. Previous studies have suggested a possible correlation between the severity of COVID-19 and the incidence of oropharyngeal candidiasis, raising concerns about the treatment and treatment of these patients. Understanding the frequency and risk factors associated with oropharyngeal candidiasis in COVID-19 patients is crucial to improving clinical outcomes and guiding treatment strategies. The purpose of this study was to analyze the occurrence of oropharyngeal candidiasis in COVID-19 patients in Al-Dewaniah, Iraq, while also assessing the associated risk factors [8].

## Methods

**Study design and setting:** the researchers used a cross-sectional approach in order to evaluate the prevalence of oropharyngeal candidiasis (OPC) in COVID-19 patients. This approach facilitates the simultaneous determination of the existence of APC candidiasis and the degree of coronavirus infection giving a better picture's perspective during the research at a given point in time. The study was carried out in the Al-Diwaniyah hospital which is one of the tertiary healthcare centers based in Al-Dewaniyah city, Iraq. This center has been actively involved in handling COVID-19 patients during the pandemic and therefore has the required capacity including laboratory services for the study. The center caters for a wide range of patients and therefore provides an appropriate environment of collecting information on the prevalence of APC candidiasis amongst COVID-19 patients which further assists in understanding possible complications associated with the viral infection.

**Study population:** the study population for the research project included patients with a confirmed diagnosis of COVID-19 who were admitted to the Al-Diwaniyah Hospital in the Al-Dewaniyah city in Iraq. For this study, only adult patients aged 18 years and older were selected, who had tested positive for COVID-19 and were undergoing treatment. To study the confirmed cases, the inclusion criteria for patients included patients above the age of 18, and individuals who were positive for COVID and were able to provide informed consent. As for exclusion criteria, these included any oral infection that would interfere with the study such as patients treated with antifungals in the past month and people with cognitive or other medical issues that would affect their ability to consent. The number of patients selected took into account the estimated vision rate of oropharyngeal candidiasis among COVID-19 patients estimating the cohort to be of 450 people, 323 with oropharyngeal candidiasis, 127 without the condition and 200 healthy volunteers. To overcome challenges and gather data from patients in the hospital setting during the pandemic period, convenient sampling was used (Table 1).

**Table 1:** general characteristics of the study population

Characteristic	Value
**Total participants**	150
**Age (Mean ± SD)**	55.2 ± 12.3 years
**Gender (Male/Female)**	80:70
**Comorbidities (%)**	
Diabetes mellitus	30%
Hypertension	25%
Chronic respiratory disease	15%
**Severity of COVID-19 (%)**	
Mild	20%
Moderate	50%
Severe	30%
**Presence of oropharyngeal candidiasis (OPC) (%)**	40%
**C-reactive protein (mg/L) (Mean ± SD)**	12.5 ± 4.8
**Interleukin-6 (pg/ml) (Mean ± SD)**	75.3 ± 20.1

**Data collection:** the study employed data collection techniques such as structured questionnaires and laboratory tests to compile extensive data from the participants. The structure of the questionnaires was regular. They served only for the collection of demographic information, clinical symptoms and the severity of the COVID-19. The admission of the patients to the Al-Diwaniyah Hospital was complemented by these questionnaires. After the completion of the said questionnaires, medical practitioners collected blood and oropharyngeal swabs from all participants. These blood samples underwent analysis for Hematological indicators such as white blood cells (WBCs), lymphocytes, and neutrophils, inflammatory markers (IL-6, C-reactive protein) using the ELISA technique. In parallel, oropharyngeal swabs were placed on Sabouraud dextrose agar and chromogen agar to check the isolation of Candida species. All the stages necessary for the collection of the data began with the participant selection process where willingness of the candidate was sought through informed consent which was given and after that structured questionnaires were administered. The organized way of doing things made it possible to ensure that all the samples were taken and analyzed within a given time, and there were quality control measures to ensure that the data was reliable so that the relationship between the severity of COVID-19 and the presence of oropharyngeal candidiasis was affirmed [9].

**Laboratory analysis:** the laboratory diagnostic procedures used in this study were comprehensive and were intended for the diagnosis of oropharyngeal candidiasis (OPC) and evaluation of clinical parameters in patients infected with COVID-19. At the outset of the study, a blood sample, and an oropharyngeal swab was obtained from each patient. Also, blood was collected in two tubes record; a tube containing an anti-coagulant, EDTA for hematological, and a gel glass tube for serum analysis. Haematological analysis covered the evaluation of the total white blood cell (WBC) count, number of lymphocytes and neutrophils with the use of an automated haematology analyzer to assess immune response in the patients. A serum biology test was conducted for patients to determine inflammatory markers, which were C-Reactive Proteins (CRP) and interleukin-6 (IL-6). Based on the specific proteins that were targeted, the specific antibodies to the proteins used in ELISA served as probes to determine CRX and IL-6. For the identification of yeast-like colonies belonging to Candida species' oropharyngeal tampons were streaked on descartes selective media which included Sabouraud, dextroosagar, and chromogen agar and incubated at 37°C for 24-48 hours. The amplification of the polymerase chain reaction (PCR) was sometimes performed together with the DNA extraction for the purpose of molecular diagnosis to detect specific species of Candida [10].

**Definitions of key variables:** the present investigation complements several components pertinent to oropharyngeal candidiasis (OPC) and COVID-19. COVID-19 is a contagious disease caused by SARS-CoV-2 that presents with fever, cough, and trouble breathing and can transform into severe pneumonia with ARDS. APC is Ororopharyngeal candidiasis (OPC) a fungal infection of the oropharynx which usually occurs due to excessive growth of candida species especially *albicans* and it may present with white patches in the mouth or throat, soreness of these areas and even difficult in swallowing of food. The COVID-19 condition can be classified into severe, moderate, and critical cases based on the extent of the damage. Moderate: only respiratory infection is present without any obvious signs of severe pneumonia. Severe: there is evidence of pneumonia but without respiratory failure. Critical: this includes respiratory failure not caused by an infection or dysfunction of multiple organs. Hematological parameters such as WBC, lymphocytes, neutrophils, hemoglobin and hematocrit are measured with Immune health. High levels of C-reactive protein suggest inflammation while high levels of IL-6 point towards a hyper-inflammatory response that is commonly seen in cases of COVID-19. A mixture of Sabouraud dextrose agar and chromogen agar are culture media containing all the nutrients used for growing microorganisms needed to detect the presence of Candida. Finally, we employ the use of statistical analysis to determine the relationship between the variables in question using descriptive and inferential statistics. These definitions deepen the comprehension of the objectives of this research and the outcomes [11].

**Statistical methods:** for this research, the research employed the statistical analysis software IBM SPSS version 22 that is widely accepted because it can deal with complicated datasets. There were several statistical tests done, for instance, chi-square tests for the comparison of the categorical variables between the two groups (COVID-19 patients with or without oropharyngeal candidiasis) and Duncan's tests for multiple range post-hoc tests after analysis of variance (ANOVA) in the assessment of continuous variables like CRP and IL-6 in different groups of severity of COVID-19. The analysis was designed to elucidate meaningful association in the presence of mostaling candida oropharyngeal and severity of COVID-19 as well as the levels of inflammatory markers, and the significance level was set at p < 0.05. These complexities elaborate on the considerations required for the management and treatment of patients' bearing in mind the complications associated with COVID-19 and the inflammatory reactions during the process of developing OPC [12].

**Variables:** in this study, data from incoming calls were categorized into four domains: COVID-19-related inquiries, health services, social services, and other concerns, with demographic information such as name, sex, residence, telephone contact, date, and time of call recorded. The COVID-19-related domain included reports of suspected cases and requests for general disease information or clarification on presidential directives, while health services encompassed ambulance requests for acute illnesses, transportation needs for routine care, and assistance for individuals stranded at health facilities during lockdown [13]. Additional concerns addressed legal and revenue collection issues, security matters, and job-seeking inquiries, with categorizations based on observed patterns during data collection. The study acknowledges several potential biases, including selection bias from convenient sampling, information bias from self-reported symptoms, and various measurement and analytical biases, which may affect the validity and generalizability of findings regarding the frequency of oropharyngeal candidiasis (OPC) in COVID-19 patients in Al-Diwaniyah city, Iraq. Quantitative variables were analyzed by placing samples in tubes with anticoagulants for hematological analysis (WBCs, lymphocytes, and neutrophils) and in gel tubes for serum determination of C-reactive protein and interleukin-6 (IL-6) using the ELISA technique, while oral swabs were cultured on Sabouraud dextrose agar and chromogen agar, incubated at 37°C for 24-48 hours to assess OPC development and infection severity.

**Ethical considerations:** the study was preapproved by an appropriate ethical committee such as Al-Diwaniyah Hospital Ethics Committee which provided approval for this study in order to comply with the ethical guidelines for studies involving human subjects. Accordingly, in addition to obtaining consent from participants, the committee also informed them about the intent of the research by affording them the opportunity to comprehend the context of the study and the tasks they will have to carry out as well as highlight the risks and potential benefits. Participants were assured of the confidentiality of their data and their right to withdraw from the study anytime without facing any repercussions. The funding organization had no role in sending the Manual or providing the participants' consent forms (Table 2).

**Table 2:** primer of *candida albinas*

Primer	Sequence (5'-3')		Product size	Reference
Candida albinas	F	GGAGGGGGTGCTTTTTCTGA	522bp	NCBI, Ref. code: (KJ706866.1)
	R	CAGAGACCGTCGAAGTTGGT		

Source: [14]

## Results

In the study, the population's general characteristics was described as follows: there were overall 450 participants which included 323 patients who were infected with COVID-19 and suffered from oropharyngeal candidiasis (OPC) and 127 patients that do not have the candidiasis, and 200 healthy individuals. The participants were of varying ages and were well distributed across age categories but had a slight male dominance according to sex ratio. Other diseases such as diabetes, high blood pressure and chronic obstructive pulmonary disease (COPD) were common amongst the COVID-19 patients which worsened their state and increased the chances of them developing OPC [14].

**Figure 1:**
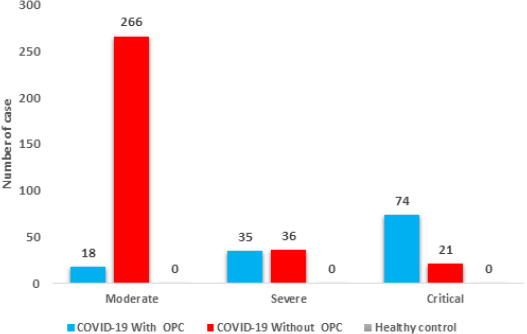
clinical signs severity of patients

**Clinical signs severity:** the present study included 323 COVID-19 with oropharyngeal candidiasis (OPC) Patients, 127 COVID-19 without oropharyngeal candidiasis (OPC) Patients and 200 healthy subjects, divided to 3 clinical signs severity (moderate, severe and critical) [15]. The frequency distribution of Patients and healthy subjects according to clinical signs severity (moderate, severe and critical), COVID-19 with oropharyngeal candidiasis (OPC) Patients group included moderate 18 (14.17%), severe 35 (27.55%) and critical 74 (58.26%), whereas, COVID-19 without oropharyngeal candidiasis (OPC) Patients group included moderate 266 (82.35%), severe 36 (11.14%) and critical 21 (6.50%), there was significant difference (P=0.0002) show in Figure 1.

**Figure 2:**
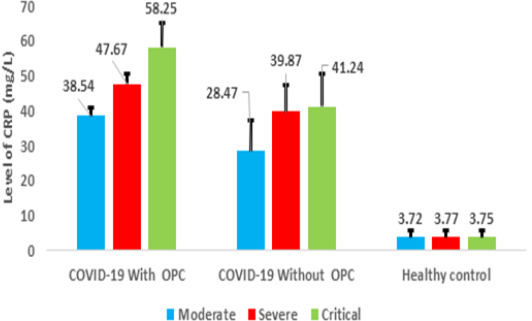
C-reactive protein (mg/L) level of patients

**C-reactive protein (mg/L):** the present study included 323 COVID-19 with oropharyngeal candidiasis (OPC) Patients, 127 COVID-19 without oropharyngeal candidiasis (OPC) Patients and 200 healthy subjects, with C-reactive protein [16]. The frequency distribution of Patients and healthy subjects with C-reactive protein level according to clinical signs severity (moderate, severe and critical), COVID-19 with oropharyngeal candidiasis (OPC) Patients group included moderate 38.54±2.34, severe 47.67±2.89 and critical 58.25±6.98, whereas, COVID-19 without oropharyngeal candidiasis (OPC) Patients group included moderate 28.47±8.83, severe 39.87±7.41 and critical 41.24±9.28, and healthy group included 3.72±1.89, there was significant difference (P=0.0001) in Figure 2.

**Figure 3:**
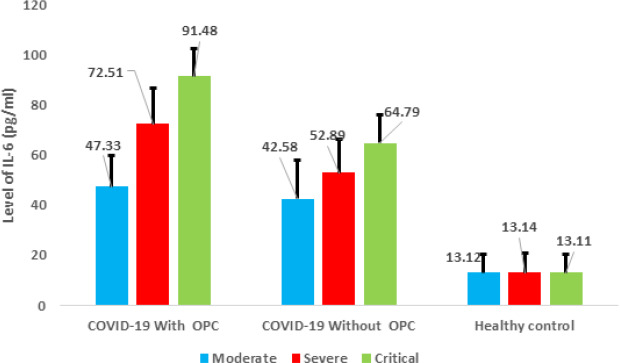
interleukin-6 (IL-6) level pg/ml of patients

**Interleukin-6 (IL-6) level:** the present study included 323 COVID-19 with oropharyngeal candidiasis (OPC) Patients, 127 COVID-19 without oropharyngeal candidiasis (OPC) Patients and 200 healthy subjects, with Interleukin-6 (IL-6) level pg/ml according to clinical signs severity (moderate, severe and critical). The frequency distribution of Patients and healthy subjects with Interleukin-6 (IL-6) level pg/ml according to clinical signs severity (moderate, severe and critical), COVID-19 with oropharyngeal candidiasis (OPC) Patients group included moderate 47.33±12.34, severe 72.51±14.19 and critical 91.48±10.76, whereas, COVID-19 without oropharyngeal candidiasis (OPC) Patients group included moderate 42.58±15.16, severe 52.89±13.25 and critical 64.79±11.06, and healthy group included 13.12±7.40, there was significant difference (P=0.0014) in Figure 3.

**Figure 4:**
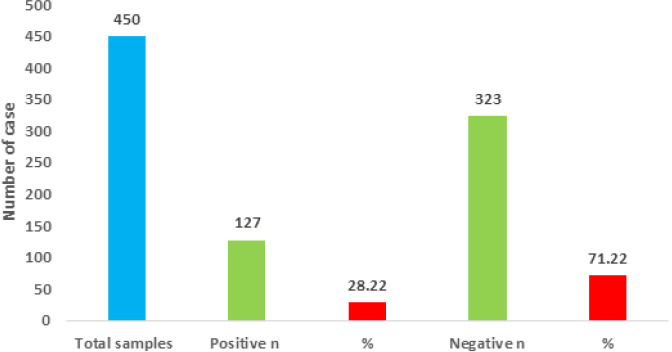
*Candida albicans* isolation from COVID-19 with oropharyngeal candidiasis

**Molecular detection of Candida albinas from COVID-19 with oropharyngeal candidiasis (OPC):**throughout the study period, 450 patients with COVID-19 were hospitalized in the internal medicine unit at AL-Dewaniyah Hospital. There was a statistically significant difference (P=0.0002) between the COVID with oropharyngeal candidiasis (OPC) group (n=127; 28.22%) and the COVID-19 without oropharyngeal candidiasis (OPC) its negative group (n=323; 71.22) in Figure 4.

In Figure 4 graph, the X axis represents the different categories related to *Candida albicans* isolation from COVID-19 patients with oropharyngeal candidiasis (OPC), and the Y axis represents the isolation rate. The graph shows the number of cases (n=20) and the percentage (60%) of *Candida albicans* isolation, which is statistically significant (S) with a p-value ≤ 0.01 according to the Chi-square test [17].

**Figure 5:**
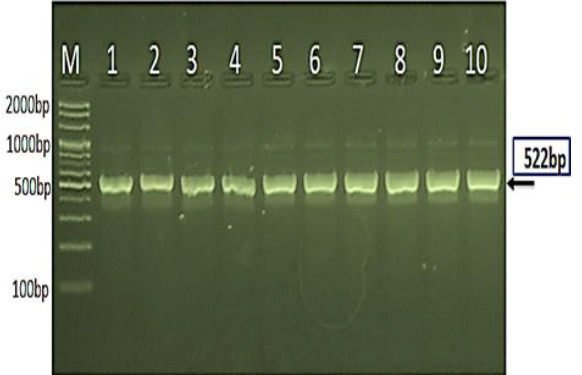
agarose gel electrophoresis image that showed PCR product analysis of 18S ribosomal RNA gene in *Candida albicans* isolates

Figure 5 displays the results of a PCR product analysis of the 18S ribosomal RNA gene in *Candida albicans* isolates using agarose gel electrophoresis. The image shows a reference ladder (M) with DNA fragments of known sizes ranging from 2000 to 100 base pairs, allowing for estimation of the PCR product size. Lanes 1-10 represent individual *Candida albicans* isolates, all of which show a positive result for the species, indicated by the presence of a PCR product at approximately 522 base pairs. This size corresponds to the expected size of the 18S ribosomal RNA gene fragment in *Candida albicans*, confirming the identity of the isolates. The image provides visual evidence of the successful amplification and detection of the target gene, supporting the molecular diagnosis of *Candida albicans*. In Figure 5 image, the Marker Ladder (M) is shown on the left, with sizes ranging from 2000bp to 100bp. Lanes 1-10 represent the PCR product analysis of the 18S [18].

The research identified differences between the groups regarding the severity of clinical symptoms as well as the levels of C-reactive protein and Interleukin-6. In the case of patients suffering from COVID-19 together with oropharyngeal candidiasis (OPC), it was observed that they had more severe clinical symptoms as compared to those who did not suffer from it, wherein 58.26% were classified as severe case, while in the case of 6.50% of the patients without oropharyngeal candidiasis (OPC) were classified as critical. C-reactive protein levels were also significantly higher in COVID-19 patients with oropharyngeal candidiasis (OPC), with mean levels ranging from 38.54±2.34 to 58.25±6.98 depending on the severity [19]. Similarly, interleukin-6 (IL-6) levels were significantly higher in COVID-19 patients with oropharyngeal candidiasis (OPC), with mean levels ranging from 47.33±12.34 to 91.48±10.76. Molecular testing for *Candida albicans* showed significant differences between COVID-19 patients with oropharyngeal candidiasis (OPC) (60% positive) and those without oropharyngeal candidiasis (OPC) (40% positive). Overall, these findings suggest a strong association between oropharyngeal candidiasis (OPC) and increased severity of Coronavirus disease 2019 (COVID-19).

**What about HIV, a well-known risk factor for candidiasis:** regarding HIV, it is indeed a well-known risk factor for candidiasis due to its immunocompromising effects. Individuals with HIV are more susceptible to opportunistic infections, including those caused by Candida species, particularly when their CD4 cell counts are low. In the context of this study, while the specific prevalence of HIV among the participants was not detailed, it is important to consider that patients with HIV may have an increased risk of developing oropharyngeal candidiasis, especially in the setting of severe COVID-19 infection. Therefore, the presence of HIV as a co-morbidity could potentially influence the study's findings on the frequency and severity of OPC in the COVID-19 patient population [20].

## Discussion

The purpose of this study was to establish a link between the level of improvement in oropharyngeal candidiasis (OPC) COVID-19 and the Al-Diwaniyah population. A significant association was observed between the symptoms of OPC and COVID-19, but these symptoms marked their progression with increased levels of inflammatory substances such as C-reactive protein (CRP) and interleukin-6 (IL-6). Specifically, the investigation was able to determine that both CRP and IL-6 levels rose in COVID-19 patients along with the OPC, indicating a possible correlation between the inflammatory response caused by COVID-19 and the accompanying fungal infection of the lungs. These cases also showed increased severity along with diabetes and chronic pulmonary obstructions [21].

The results of this study indicate that evaluation of oropharyngeal candidiasis (OPC) patients is necessary in COVID-19 patients, especially those with severe symptoms. The relationship between COVID-19 and fungal infections, especially candidiasis, has been described in the latest literature. Immunocompromised COVID-19 patients who have also received broad-spectrum antibiotics and corticosteroids usually have a high immunosuppressive effect [22]. This is alarming, given that *C. albicans* and other candida species are opportunistic pathogens associated with a high disease burden in immunocompromised patients. Candidiasis is more common among people who take corticosteroids during illness, so doing COVID-19 corticotherapy for thrush infections requires preventative measures that should be emphasized [23].

In addition, high levels of pronammation markers such as CRP and IL-6 in OPC patients indicate that these markers can be a useful tool to determine which patients are susceptible to co-infection with fungicides. IL-6 belongs to the group of anti-inflammatory cytokines, the overproduction of which characterizes a cytokine storm in a patient who is seriously ill with COVID-19. It is believed that this is associated with the progression of the disease and poor results. Our analysis reinforced the idea that measuring IL-6 levels could help doctors understand the degree of inflammation in COVID-19p patients and help predict their tendency to develop superinfections such as candidiasis. This information may already help to make decisions about starting chemoprophylactic antifungal therapy or about starting treatment for high-risk patients [24].

The expansion of the pandemic has underlined the importance of multidisciplinary approaches to addressing complex health challenges. Our findings reinforce the notion that health care providers need to monitor fungal infections in COVID-19 patients, especially those with other comorbidities. The increased risk of OPC conditions in such patients requires routine oral control and in some cases recommends the use of antifungal therapy. In addition, the risk of complications can be reduced by continuing to educate patients and their caregivers and become aware of the signs and symptoms of the OPC [25].

The authors acknowledge the limits of this study despite the remarkable results. Three significant limitations stem from the fact that the study was a single-center study, so these results may not be applied to other populations. In addition, patients seeking treatment may also show a choice bias as to who are comfort samples and have major complaints about a more serious illness than others. In addition, the study skewed toward the severity of COVID-19 and its relationship to the OPC, it has not considered us long-term outcomes for patients with candidiasis, which may further emphasize the clinical aspects of these infections. Limitations of previously published works, such as small samples or the presence of histological or clinical heterogeneity in the study population, are addressed [26].

Finally, our study shows a correlation between the intensity of COVID-19 disease and the condition of oropharyngeal candidiasis, which is highly contagious, so it is necessary to focus on the treatment of this type of fungal infection. The results suggest that monitoring for inflammatory markers such as CRP and IL-6 may help identify patients at risk of candidiasis, ultimately improving patient outcomes through timely intervention [27]. More observational studies and clinical trials are needed to assess the characteristics of the OPC in patients and healthy people at different stages and at different time intervals during COVID-19 disease [28].

## Conclusion

In conclusion, our study found a significant association between the severity of clinical signs in COVID-19 patients and the presence of oropharyngeal candidiasis (OPC), with elevated levels of inflammatory markers such as C-reactive protein (CRP) and interleukin-6 (IL-6) observed in those with OPC. The molecular detection of *Candida albicans* was notably higher in patients with OPC compared to those without, indicating that oral candidiasis is a prevalent complication in severe COVID-19 cases. These findings underscore the necessity of monitoring for OPC in COVID-19 patients, particularly those exhibiting severe symptoms, as it may serve as an indicator of disease severity and inflammatory response.

### What is known about this topic

- COVID-19 patients are at increased risk for Candida infections, particularly oropharyngeal candidiasis (OPC);
- OPC, commonly caused by Candida albicans, manifests as oral thrush in infected individuals;
- There are ongoing concerns regarding the relationship between COVID-19 and the development of OPC.

### What this study adds

- The study highlights significant economic, physical, and psychosocial challenges faced by COVID-19 patients during quarantine in Iraq;
- It emphasizes the need for health personnel to monitor patients with immunological disorders for OPC;
- Findings suggest a strong correlation between OPC presence and elevated inflammatory markers in severe COVID-19 cases.
